# Bats reduce insect density and defoliation in temperate forests: An exclusion experiment

**DOI:** 10.1002/ecy.3903

**Published:** 2022-12-21

**Authors:** Elizabeth A. Beilke, Joy M. O'Keefe

**Affiliations:** ^1^ Department of Natural Resources and Environmental Sciences University of Illinois at Urbana‐Champaign Urbana Illinois USA; ^2^ Center for Bat Research, Outreach, and Conservation Indiana State University Terre Haute Indiana USA

**Keywords:** bats, defoliation, ecosystem services, exclosure, forests, hickories, insectivory, insects, oaks, trophic cascades

## Abstract

Bats suppress insect populations in agricultural ecosystems, yet the question of whether bats initiate trophic cascades in forests is mainly unexplored. We used a field experiment to test the hypothesis that insectivorous bats reduce defoliation through the top‐down suppression of forest‐defoliating insects. We excluded bats from 20 large, subcanopy forest plots (opened daily to allow birds access), each paired with an experimental control plot, during three summers between 2018 and 2020 in the central hardwood region of the United States. We monitored leaf area changes and insect density for nine to 10 oak or hickory seedlings per plot. Insect density was three times greater on seedlings in bat‐excluded versus control plots. Additionally, seedling defoliation was five times greater with bats excluded, and bats' impact on defoliation was three times greater for oaks than for hickories. We show that insectivorous bats drive top‐down trophic cascades, play an integral role in forest ecosystems, and may ultimately influence forest health, structure, and composition. This work demonstrates insectivorous bats' ecological and economic value and the importance of conserving this highly imperiled group of predators.

## INTRODUCTION

Trophic cascades—that is, indirect species interactions that originate with predators and spread downward through food webs (Ripple et al., 2016)—have been observed in many systems (Pace et al., 1999) and remain a topic of considerable interest in ecology. The concept of the trophic cascade first emerged to explain the presence of green vegetation on earth; herbivores do not defoliate the world because their populations are limited by the presence of predators (Hairston et al., 1960). This view is overly simple, and we now recognize that predators are more likely to influence the types of plants present at a site than the presence or absence of vegetation (Wilkinson & Sherratt, 2016). Defoliation reduces plant growth, vigor, competitive ability, and fitness, which are issues exacerbated by environmental stressors such as drought or nutrient scarcity (Gottschalk, 1989; Jedlicka et al., 2004; McGraw et al., 1990; Rieske & Dillaway, 2008; Wargo, 1996; Wright et al., 1989). Thus, by reducing defoliation, predators contribute to the structure and composition of ecosystems, and, consequently, the loss of predators leads to community reorganization and the decline of diversity within those systems (Ripple & Beschta, 2012; Terborgh, 2015; Terborgh et al., 2006).

Various attributes may modify the strength of a trophic cascade, including attributes of the organisms involved in the cascade. For example, trophic cascades are strongest in systems with highly mobile, endothermic predators with high metabolisms (Borer et al., 2005). They are also strongest in systems with invertebrate herbivores (Borer et al., 2005), especially when these animals have generalist dietary habits (Singer et al., 2014). Accordingly, highly mobile insectivorous vertebrates, such as birds and bats, are often important initiators of top‐down trophic cascades (Maas et al., 2016; Mäntylä et al., 2011; Mooney et al., 2010; Van Bael et al., 2008). Although trophic cascades are thought to be strongest in less complex ecosystems with fewer intermediate predators to attenuate the effects of top predators (Polis & Strong, 1996), this assumption is not broadly applicable to highly mobile predators like birds and bats (Borer et al., 2005). The top‐down effects of birds and bats do not vary with habitat complexity (Van Bael et al., 2008) or habitat type (tropical vs. temperate; Maas et al., 2016), and they are generally stronger in systems with more intermediate predators (Mooney et al., 2010).

Bats have several characteristics that position them as strong regulators of prey populations. For example, bats have higher mass‐specific basal metabolic rates than many other mammals (Austad & Fischer, 1991; Speakman & Thomas, 2005) and exhibit numerical and functional responses to artificially inflated prey densities (i.e., general activity and feeding rate increase with increasing prey density; Charbonnier et al., 2014). Bats also respond to and exploit natural pulses in prey populations (Cohen et al., 2020; McCracken et al., 2012), in turn reducing prey abundance (Puig‐Montserrat et al., 2015). Bats exert top‐down control on many agricultural pests, including corn earworms (*Helicoverpa zea*; Maine & Boyles, 2015), cotton bollworms (*Helicoverpa armigera*; Federico et al., 2008), and rice borer moths (*Chilo suppressalis*; Puig‐Montserrat et al., 2015). Moreover, this top‐down control is strong enough to indirectly benefit crops, such that the exclusion of bats from agricultural plots decreases crop quality and yield (Maas et al., 2013; Maine & Boyles, 2015). Because prior work demonstrated that bats caused top‐down trophic cascades regardless of habitat complexity, we predict that bats will also be shown to be essential predators in complex environments like forests.

Although most bat species rely on forests as roosting and foraging habitats, bats have typically been ignored in studies of forest defoliator control. However, bats consume various forest‐defoliating insects (Beilke, 2022; Clare et al., 2009; Divoll et al., 2022; Dodd et al., 2012), and their pest suppression likely extends to complex forest ecosystems (Mooney et al., 2010). To our knowledge, only a single study has excluded bats from forest plants to examine how bats impact forest defoliation, where tropical lowland plants from which gleaning bats were excluded experienced greater arthropod density and defoliation than control plants (Kalka et al., 2008). However, most bird exclusion studies have unintentionally excluded bats in their studies (by not opening or removing their experimental exclosures at night; e.g., Harris et al., 2020; Lichtenberg & Lichtenberg, 2002; Marquis & Whelan, 1994; Sipura, 1999; Van Bael et al., 2003). Although there is a tendency in these studies to downplay the potential impact of bats within the study area, if bats are acknowledged at all, the pest and defoliation suppression these studies attributed to birds are almost certainly shared by bats. Direct comparisons of the top‐down suppression of insects by birds and bats demonstrate that, compared to birds, bats have similar or stronger capacities to limit insect density and defoliation (Kalka et al., 2008; Maas et al., 2019; Morrison & Lindell, 2012; Williams‐Guillén et al., 2008). For example, bats in Indonesia contribute three times more to cocoa yields than birds (Maas et al., 2019), and bats in Panama reduce the density and folivory of insects on understory plants twofold relative to birds (Kalka et al., 2008). Thus, bats likely play a vital but previously overlooked functional role in forests. Ultimately, bat conservation may be essential to sustaining healthy forests, which are economically, ecologically, and culturally significant resources.

In this study, we tested the hypothesis that insectivorous bats cause trophic cascades in forests, thereby reducing insect density and forest defoliation by insects. We experimentally excluded bats from large, subcanopy plots to examine (1) how bat presence or absence affects seasonal changes in seedling defoliation, (2) whether bats have a variable effect on two genera of ecologically and economically important deciduous tree species: oaks (*Quercus* spp.) and hickories (*Carya* spp.), and (3) whether bats reduce insect density. We predicted that bat‐excluded seedlings would harbor more insects and suffer greater levels of seasonal defoliation than control seedlings. We also predicted that bats would have a more significant impact on oaks than hickories because oaks are known to host a greater diversity of lepidopterans (Narango et al., 2020; Tallamy & Shropshire, 2009), which are common prey items for bats in the area where we worked (Beilke, 2022; Divoll et al., 2022).

## METHODS

### Study area

We conducted this study in Yellowwood State Forest in south‐central Indiana, USA (39.1° N, 86.3° W). In this area, oak, hickory, and tulip poplar (*Liriodendron tulipifera*) dominate the overstory, whereas maple (*Acer* spp.) and American beech (*Fagus grandifolia*) dominate the midstory. We performed our experiments in five separate thinned stands, which were 0.4–6.8 km apart. The midstory of these stands was thinned in 2008–2009, and the overstory was thinned in 2015–2016, reducing the stand basal areas to 13.8–16.1 m^2^/ha (Kalb & Mycroft, 2013).

The bats in this system catch their prey in midair or glean prey from surfaces. According to capture records, eight bat species occur in this study area in the following order of abundance: eastern red bat (*Lasiurus borealis*), Indiana bat (*Myotis sodalis*), big brown bat (*Eptesicus fuscus*), northern long‐eared bat (*Myotis septentrionalis*), hoary bat (*Lasiurus cinereus*), tricolored bat (*Perimyotis subflavus*), silver‐haired bat (*Lasionycteris noctivagans*), and little brown bat (*Myotis lucifugus*).

The principal defoliators in this system are moths (Lepidoptera), beetles (Coleoptera), true bugs (Hemiptera), and katydids and crickets (Orthoptera). The bats that occur in the study area consume all of these groups, taking prey items with wingspans that range from 1.25 to 174 mm (Clare et al., 2009; Cravens et al., 2018; Divoll et al., 2022; Whitaker, 2004). The most common large‐bodied moth families in the study area are Erebidae, Noctuidae, Notodontidae, and Geometridae; the most common small‐bodied families are Gelechiidae, Tortricidae, and Gracillariidae (Divoll et al., 2022; Summerville et al., 2013). Among other insects, the herbivorous families we most observed in the study area and our plots were beetles (Circulionidae, Chrysomelidae, Cerambycidae, Elateridae, and Scarabaeidae), true bugs (Cicadellidae, Miridae, Pentatomidae), and katydids and crickets (Gryllidae and Tettigoniidae). The insects we frequently observed on study seedlings were small‐bodied lepidopteran larvae, small‐bodied Circulionids, Cicadellids, and Gryllids. All of these insects produce one to several generations per year.

### Experimental design

**FIGURE 1:**
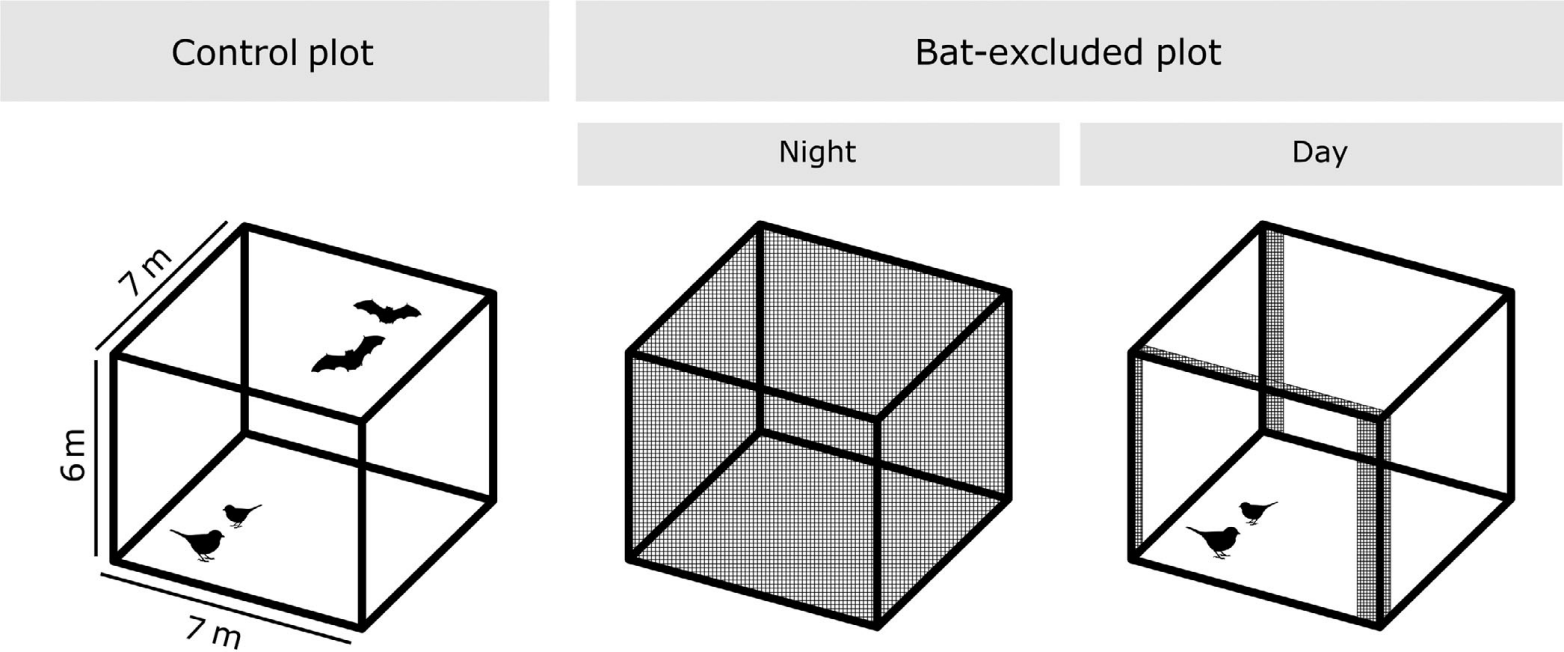
Schematic of experimental unit (*n* = 20). Each experimental unit consisted of a netless control plot and a netted bat‐excluded plot, which were deployed within 15 m of each other and considered paired for the purposes of our analyses. Bat‐excluded plots were closed at night to deny bats access and were opened during the day to allow access by birds.

We deployed 20 experimental units across a 3‐year period (seven per year; one was lost). Each experimental unit consisted of a netted exclosure (hereafter, “bat‐excluded plots”) and a netless control structure (hereafter, “control plots”), which were deployed within 15 m of each other, close enough that seedlings within the plots likely experienced similar environmental conditions and insect communities. We considered these plots paired for the purposes of our analyses (Figure 1). All experimental units were monitored from late May to mid‐August in 2018, 2019, or 2020 (hereafter, “treatment period”); this period corresponds to a surge in bat foraging activity associated with the increased caloric demands of reproduction (Barclay, 1989). In 2019 we lost one experimental unit in a storm, resulting in a final sample size of 20 experimental units over three seasons. Bat‐excluded plots were opened each morning (around 6:00 a.m. Eastern Daylight Time [EDT]) to allow birds access and closed each evening (around 7:00 p.m. EDT) to deny bats access.

### Exclosure construction

We built large exclosures that would exclude aerial hawking bats (6 × 7 × 7 m; Figure 1; for photo, see Appendix S1: Figure S1). Each structure consisted of four galvanized steel poles connected by a steel cable topline. To exclude bats, we suspended square‐knot nylon mesh netting (4.44‐cm openings) from frames using metal rings, which enabled the net to slide along the steel topline using a series of rope pulleys. The net extended all the way to the ground with excess length to drape. The mesh size of the netting was selected to exclude bats without excluding the local insect fauna. In control structures, the frames remained netless. For stability, each pole sat atop a rebar pin driven into the ground and was held upright by a pair of perpendicular guy lines. These structures were reused each summer, though moved about the landscape, for the duration of the study.

### Data collection

Within each plot, we randomly selected nine to 10 oak and hickory seedlings, irrespective of species, to monitor during the treatment period. Ultimately, our data set included 235 oak (122 treatment, 113 control) and 161 hickory seedlings (74 treatment, 87 control). Our samples were weighted in favor of oaks because they were more abundant. Oak species included, in order of abundance, *Quercus velutina*, *Q. alba*, *Q. rubra*, *Q. coccinea*, and *Q. montana*. Hickory species included, in order of abundance, *Carya ovata, C. glabra*, and *C. cordiformis*. Seedling height ranged from 4 to 166 cm (30 ± 20 cm, mean ± SD).

For seedlings with <30 leaves, we monitored all leaves, but for seedlings with >30 leaves, we used a random number generator to select branches to monitor until we had acquired a sample size of at least 30 leaves. On average, we monitored 25 ± 17 leaves per seedling (mean ± SD). We marked each leaf for horizontal tracking and quantified its leaf area at the start and end of the treatment period. This process varied in 2018 versus 2019–2020. In 2018, we visually estimated leaf area by laying a plastic 1‐cm grid over the top of each leaf, counting the number of squares occupied by green leaves and the number of squares removed by insect damage. We used these data to calculate the proportion of each leaf that was defoliated and the percentage change in those defoliation levels across the season. In 2019 and 2020, we photographed all study leaves at the start and end of the season and quantified their green leaf area using Easy Leaf Area, a software program that calculates green leaf area relative to a red square of a known area (Easlon & Bloom, 2014). We used these estimates of leaf area to calculate the percentage change in leaf area across the season. Both methods excluded dead or discolored portions of the leaves from our leaf area estimates, thereby accounting for damage by insects that affect leaves without removing leaf tissue (e.g., skeletonization, sucking, mining, or galling). We performed both techniques on a subset of leaves (*n* = 6) and found no significant differences in the defoliation estimates they yielded (two‐way, paired *t*‐test: *t* = 0.99, df = 5, *p* = 0.923). Thus, we pooled these data for downstream analysis. We define “defoliation” as the percentage change in leaf area from the beginning to the end of the treatment period (per seedling).

We surveyed insect abundance on every study seedling at two time points each year—at the beginning and end of the treatment period. These surveys occurred in the early afternoon. To quantify insect abundance, we carefully inspected each study seedling, including the tops and bottoms of every leaf, noting how many insects were present. Infrequently, we found insect eggs during our surveys. We noted those separately. Owing to constraints on time and expertise of myriad insect taxa and morphs, we did not identify insects to the species level in the field.

We surveyed bat activity at each experimental unit (using acoustic bat detectors) and periodically monitored nonbat activity within each plot (using trail cameras). Bat activity did not vary significantly with experimental unit, and we recorded very few instances of nonbat fauna entering plots. Thus, we did not include these variables in our analyses. We present these methods and the resulting data in our supplementary appendix (Appendix S1: Sections S1 and S2, Table S1 and Figure S2).

### Reliability analyses

We examined the reliability of each previously described technique for a subset of our study leaves (*n* = 12). To assess test–retest reliability or measurement error, we photographed each leaf three times, quantified the leaf area of each photo, and tested how closely these leaf area estimates resembled each other (Yen & Lo, 2002). To assess intrarater reliability or intraobserver bias, we quantified the area of each leaf three times and examined how closely those estimates resembled each other. Intraclass correlation estimates were calculated using the irr package (Gamer et al., 2019) in R (R Core Team, 2021) and were based on a mean‐rating, absolute‐agreement, two‐way mixed model (Koo & Li, 2016). Reliability was high in all cases (intraclass correlation coefficient >0.99).

### Statistical analysis

We examined the relationship between defoliation, bat exclusion, and tree genus using linear mixed models. We constructed a full model that predicted defoliation as a function of treatment (control vs. bat‐excluded), tree genus (oak vs. hickory), and the interaction between treatment and tree genus. The full model also included a year covariate (2018, 2019, or 2020) to account for annual variation and a random effect that accounted for the nesting of trees within experimental units. Therefore, the full model was defoliation ~ treatment × tree genus + year + (1|experimental unit/tree ID). To assess whether treatment, tree genus, and the interaction between the two improved the model, we compared the AIC_c_ value for the full model to a reduced null model containing only the covariate and random effects (defoliation ~ 1 + year + [1|experimental unit/tree ID]) (Forstmeier & Schielzeth, 2011). This model selection approach is useful for testing explicit a priori hypotheses and reduces the likelihood of making a false‐positive error relative to approaches that consider many models (Tredennick et al., 2021). We assessed the fit of the full model by checking its residual plots and calculated confidence intervals (CIs) around predicted relationships, incorporating fixed‐effects uncertainty only, using a parametric bootstrap approach with 500 resamples. Model residuals were normally distributed and exhibited no clear patterns (i.e., heteroscedasticity). We evaluated parameter significance at *p* < 0.05 and expressed uncertainty in model predictions with 95% CIs.

To investigate the relationship between insect density and bat exclusion, we constructed a generalized linear mixed model with experimental unit ID as a random intercept and a negative binomial family to account for overdispersion in the data. Our model included experimental treatment (control vs. bat‐excluded), time point (initial vs. final), and the interaction between experimental treatment and time point as fixed effects (insect density per plant ~ experimental treatment × time point + [1|unit ID]).

All analyses were performed in R version 4.1.1 (R Core Team, 2021) and RStudio version 1.4.1717 (RStudio Team, 2021). We used the lme4 package to fit mixed models (Bates et al., 2015), the DHARMa package to evaluate model fit (Hartig, 2021), and the lme4 and boot packages to calculate CIs (Bates et al., 2015; Canty & Ripley, 2021). We produced figures using the ggplot2 and patchwork packages and used Inkscape version 1.1.1 for postproduction (Inkscape Project, 2021; Pedersen, 2020; Wickham, 2016).

## RESULTS

**FIGURE 2:**
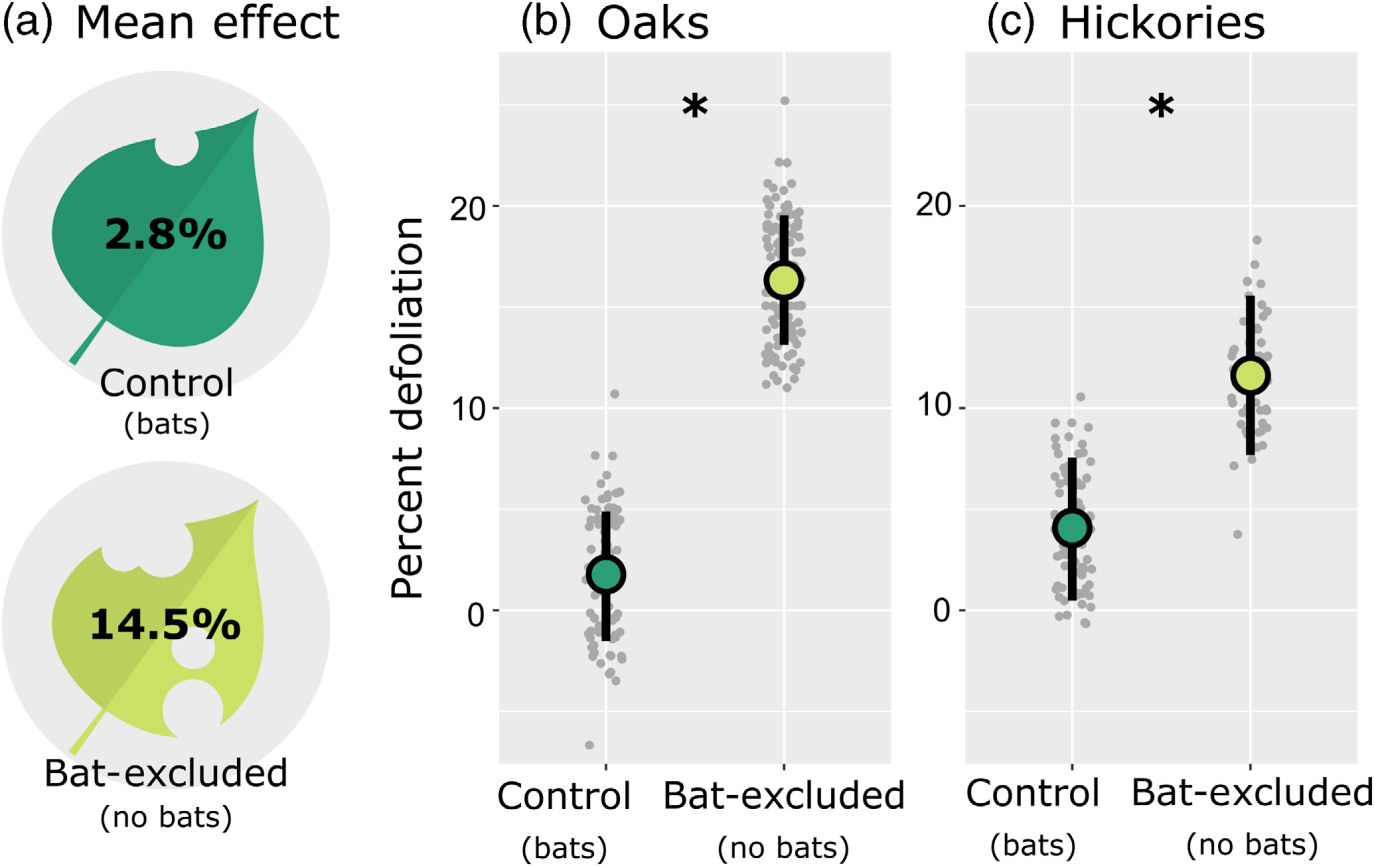
Excluding insectivorous bats from forest subcanopy plots led to greater insect defoliation (a) of the oak ([b] 1.8% in control vs. 16.3% in bat‐excluded plots) and hickory ([c] 4.1% vs. 11.6%) seedlings within. To visualize the mean leaf area removed in control (*n* = 20) and bat‐excluded plots (*n* = 20), we calculated the area of our leaf icons and removed an appropriate amount (a). The relationships between percentage defoliation (percentage of initial leaf area lost across treatment period), experimental treatment, and tree genus are portrayed in (b) and (c). Gray points (*n* = 396) represent fitted data values, enlarged bubbles portray fitted means, and vertical bars represent 95% confidence intervals (calculated using bootstrap resampling with 500 simulations). Fitted values come from a linear mixed model (see section Statistical analysis).

Defoliation was higher where bats were excluded, and oak seedlings were more affected by bat exclusion than hickories (Figure 2). Our full model outperformed the null model (null ΔAIC_c_ = 60.83), carrying ~100% of the model weights, and both treatment and the interaction between seedling genus and treatment were significant predictors of defoliation (*F*_1,395_ = 69.64, *p* = 0.001 and *F*_1,395_ = 5.26, *p* = 0.022, respectively; Table 1). On average, bat‐excluded seedlings experienced five times more defoliation than control seedlings (14.5% vs. 2.8% defoliation in bat‐excluded vs. control plots; Figure 2a). Oaks experienced nine times more defoliation in plots from which bats were experimentally excluded (Figure 2b; 16.3% vs. 1.8% defoliation in bat‐excluded vs. control plots), whereas hickories experienced three times more defoliation in experimental plots (Figure 2c; 11.6% vs. 4.1% defoliation in bat‐excluded vs. control plots). Genus alone (oak vs. hickory) was not a significant predictor of defoliation (*F*_1,395_ = 1.61, *p* = 0.193; Table 1).

**TABLE 1:** Experimental treatment (bat‐excluded vs. control) and the interaction between experimental treatment and seedling genus (oak vs. hickory) were significant predictors of seedling defoliation levels.

Predictor	Estimate	95% CI	*p*
(Intercept)	**4.29**	**0.66 to 7.91**	**0.020**
Genus (oak)	−2.66	−6.66 to 1.35	0.193
Treatment (bat‐excluded)	**7.76**	**3.35 to 12.17**	**0.001**
Year (2018 vs. 2019)	3.20	−0.40 to 6.79	0.081
Year (2018 vs. 2020)	−2.87	−6.31 to 0.56	0.101
Year (2019 vs. 2020)	**−6.07**	**−9.72 to −2.42**	**0.001**
Genus (oak) × treatment (bat‐excluded)	**6.74**	**0.98 to 12.50**	**0.022**

*Note*: Values come from a linear mixed model (formula = defoliation ~ treatment **×** tree genus + year + [1|experimental unit/tree ID]). Reference level for genus, treatment, and year are hickory, control, and 2018, respectively. Significant variables (*p* < 0.05) are bolded.

**FIGURE 3:**
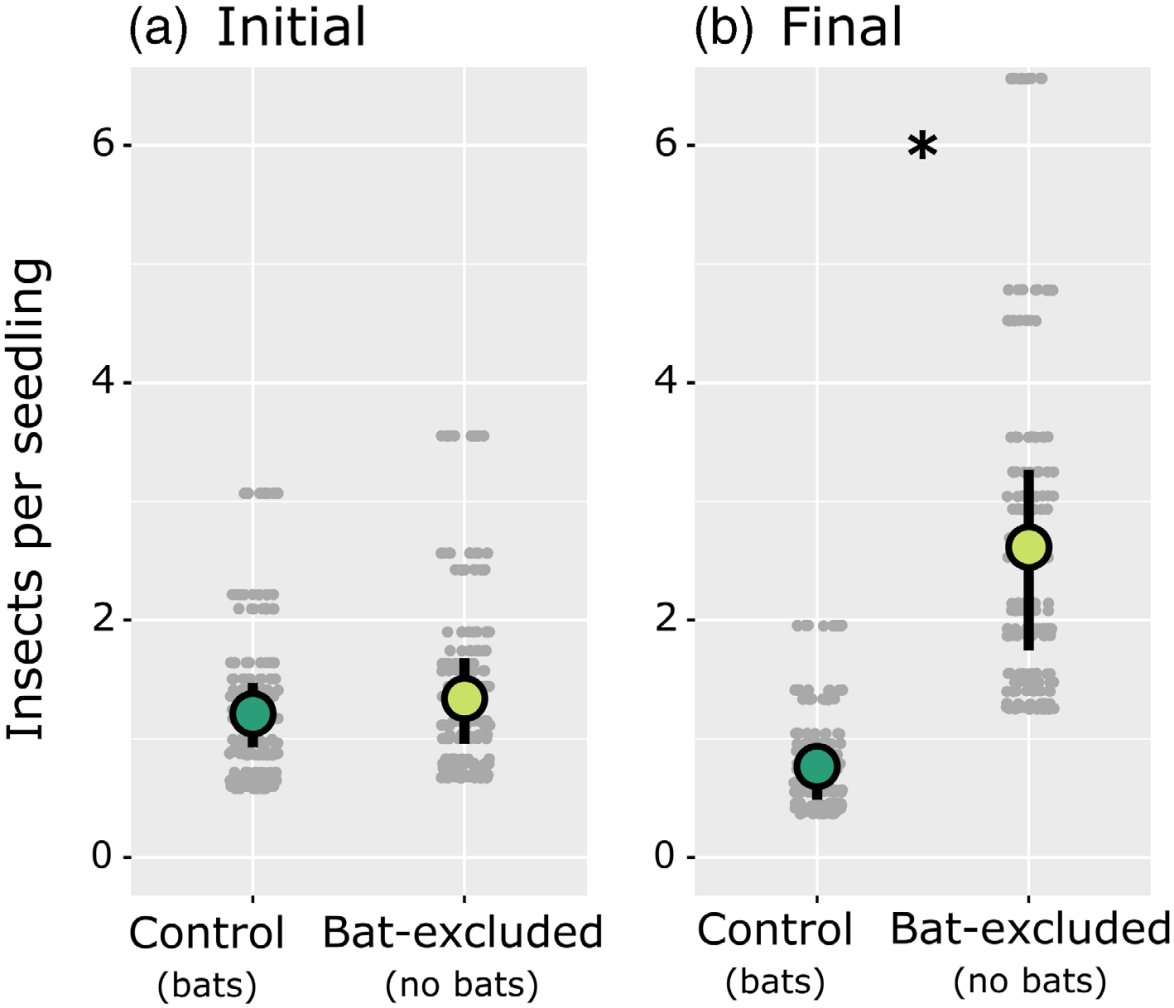
Insect density (model‐fitted number of insects per study seedling) declined in control plots but increased in bat‐excluded plots from the beginning ([a] 1.2 insects in control plots and 1.4 insects in bat‐excluded plots) to the end ([b] 0.8 and 2.6 insects) of the treatment period. Gray points (*n* = 792) represent fitted data values, enlarged bubbles portray fitted means, and vertical bars represent 95% confidence intervals (calculated using bootstrap resampling with 500 simulations). Fitted values come from a linear mixed model (see section Statistical analysis).

At the beginning of the treatment period, insect density (the number of insects per study seedling) did not differ between control and bat‐excluded seedlings (*z*_1,791_ = 0.90, *p* = 0.370; Figure 3). By the end, insect density was significantly higher in bat‐excluded plots than in control plots (*z*_1,791_ = 21.93, *p* ≤ 0.001; Figure 3). Across the treatment period, on average, insect density decreased by 0.4 insects per seedling in control plots (*z*_1,791_ = −2.66, *p* = 0.008) and increased by 1.2 insects per seedling in bat‐excluded plots (*z*_1,791_ = 3.97, *p* ≤ 0.001; Figure 3). We made six times as many observations of groups of >10 lepidopteran larvae on bat‐excluded seedlings versus control seedlings (for example photo, see Appendix S1: Figure S3). In total, we noted 122 versus 19 insect eggs on bat‐excluded versus control seedlings in our final surveys.

## DISCUSSION

We excluded bats from forest subcanopy plots to quantify the indirect effect their presence had on seedling defoliation. By comparing bat‐excluded plots to paired control plots, we demonstrated that bats reduced seedling defoliation fivefold. In addition, we showed that bats reduced oak defoliation threefold relative to hickories. Bats also reduced insect density threefold. These results demonstrate that insectivorous bats may play a crucial role in structuring forest ecosystems. If so, forests may be impacted by global bat declines (Frick et al., 2020); hence, this work further emphasizes the need to conserve bat populations.

Bat‐excluded plants experienced significantly more defoliation than control plants. The defoliation levels we observed in control and bat‐excluded plants were similar to those reported for bats in tropical forests (Kalka et al., 2008) and temperate cornfields (Maine & Boyles, 2015). Previously, bats had been overlooked as top‐down suppressors of insects in temperate deciduous forests, particularly in bird research. Bird exclusion studies, which tend to use permanent 24‐h exclosures, have confused the ecological impact of flying insectivores for the impact of birds alone (Maas et al., 2019). Distinguishing the top‐down impact of birds versus bats is particularly important because both groups are declining (Frick et al., 2015; Rosenberg et al., 2019). Currently, we do not know the extent to which the diets of sympatric birds and bats overlap, nor do we understand whether the two groups are functionally redundant. In the future, bird‐exclusion studies should open their exclusion structures at night to allow bats access to their treatment plots or plants. Where resources allow, it is also advisable to directly compare the top‐down impacts of birds and bats (e.g., Kalka et al., 2008).

The discrepancies in defoliation levels between seedlings in bat‐excluded and control plots were greater for oaks than hickories, indicating that oaks receive a greater benefit from bats' presence than hickories. It is possible that bats are consuming more oak defoliators than hickory defoliators. We compiled a list of the prey items (*n* = 321) included in the diet of eastern red bats (Clare et al., 2009; Cravens et al., 2018), the most commonly captured species in our study area, and searched BugGuide.net for information about the host plants of those insects. This cursory investigation revealed that eastern red bats consume three times more oak‐defoliating than hickory‐defoliating insect species. These results suggest bats may affect the composition of forest canopies by conferring a competitive advantage on some tree species. If so, forests could be altered by the ongoing decline of bats from the region (Pettit & O'Keefe, 2017), and bat declines could be one of the many factors contributing to oak declines in the eastern United States (Haavik et al., 2015).

The levels of defoliation we observed were likely sublethal since we observed few mortality events during our study. However, the loss of 14.5% of leaf area could reduce the overall growth or competitive ability of seedlings or result in mortality in the presence of synergistic factors. For example, the availability of sunlight, water, and nutrients interacts with defoliation to influence oak seedling photosynthesis, growth, and survival (McGraw et al., 1990). During a drought, even low levels of defoliation cause mortality in northern red oak seedlings (*Q. rubra*; Wright et al., 1989). Furthermore, defoliation by insects makes plants more susceptible to disease since insects are common vectors of plant pathogens (Eigenbrode et al., 2018), and previous research demonstrated that bats, by suppressing insect populations, reduced the prevalence of insect‐associated pathogens (Maine & Boyles, 2015). Given broad declines in aerial insectivores (Frick et al., 2020; Rosenberg et al., 2019), it is worth examining how different tree species cope with persistent sublethal defoliation, especially given the role insects and pathogens may play in structuring ecosystems (Orwig, 2002). Such research could clarify how dynamic populations of insectivorous predators might ultimately influence forest structure and composition.

Bats likely benefit plants directly by reducing insect density and indirectly by altering insect behavior. In addition to consuming insects that defoliate plants, bats may further benefit plants by consuming adult insects that produce offspring that defoliate plants. This is an important distinction because some of the insects bats eat do not defoliate in their adult phase (e.g., lepidopterans). We noted more insect eggs in bat‐excluded plots, and others have observed relationships between bat activity and insect reproductive success (Charbonnier et al., 2014; Maine & Boyles, 2015). Thus, it is likely that bats protect plants even by consuming adult insects that do not defoliate plants themselves. Although much of this protection probably occurs directly, bats may also protect plants by creating an acoustic landscape of fear that alters the behavior of their prey (Laundré et al., 2001). Auditory cues from predators influence insect behavior. For example, batlike ultrasonic pulses reduce ovipositing by cabbage looper moths (*Trichoplusia ni*; Payne & Shorey, 1968), and wasp buzzing reduces development time and final pupal weight in monarch caterpillars (*Danaus plexippus*; Lee et al., 2021).

By design, exclusion studies have limitations, but we are confident that we accounted for many of them. For example, our nets may have excluded the largest insects in our study area. However, we observed insects as large as the eastern tiger swallowtail (*Papilio glaucus*; wingspan up to 14 cm) flying through these nets and concluded that only very large lepidopterans (e.g., luna moth, *Actias luna*; wingspan up to 17.8 cm) might have been excluded. Additionally, our bat‐excluded plots necessarily excluded other large‐bodied nocturnal animals, including some that may eat plants or insects (for a summary, see Appendix S1: Table S1). However, our trail cameras recorded few visits by large‐bodied animals (Appendix S1: Table S1), suggesting they did not impact this study. We did not formally survey bird activity, but we flushed Carolina wrens (*Thryothorus ludovicianus*) and worm‐eating warblers (*Helmitheros vermivorum*) from both control and bat‐excluded plots on multiple occasions. Therefore, we are confident that birds used experimental units during the day. We also know from our passive acoustic surveys that bats were using the experimental units extensively throughout the study and were always present in the landscape (Appendix S1: Figure S3).

This work prompted several exciting follow‐up questions that could deepen our understanding of the relationships between bats, insects, and plant communities. For example, how does forest composition affect the quality of bat food supplies and, in turn, bat health? Our research suggests a strong connection between oaks, oak‐defoliating insects, and bats. Thus, changes in forest composition (e.g., oak declines) could reduce the quality of bat foraging habitat and, in turn, the health of bat populations. Additionally, what is the significance of natural ecosystems in providing highly modified landscapes with bat‐provisioned ecosystem services? Although bats may forage over agricultural fields, most species still rely on trees, woodlots, or forests for cover (e.g., Sparks et al., 2005). Thus, proximity to forests is likely a strong indicator of bat‐initiated pest suppression in modified landscapes (Heim et al., 2017; Treitler et al., 2016), but the literature has largely ignored this detail (Fisher & Naidoo, 2011). Lastly, our research strongly suggests that bats play a role in structuring forest ecosystems, but more work is needed to understand the extent to which this is true. Admittedly, it would be challenging to disentangle the role of bats from the myriad other influences on ecosystem structure and function. However, bottlenecks in bat populations caused by stressors such as white‐nose syndrome, a fungal disease that has decimated populations of hibernating bats in North America (Hoyt et al., 2021), could provide an excellent opportunity to study this phenomenon as a natural experiment. Changes in bat community composition and relative abundance might be linked to long‐term changes in the fitness or relative abundance of different tree species.

In conclusion, bats' ecological role in forest ecosystems has long been overlooked. In a novel experiment, we demonstrated that bats reduced insect density and the defoliation of oaks and hickories in temperate deciduous forests, indicating that bats likely influence the health, structure, and composition of forests. Given the broad global distribution of insectivorous bats, this relationship likely extends to many other ecosystems, and we must endeavor to conserve these essential but highly imperiled predators. Ultimately, this research adds to the sparse literature addressing the impact of bats on forest ecosystems and highlights the ecological and economic value of bats and the importance of their conservation to humans and nonhumans alike.

## AUTHOR CONTRIBUTIONS

Conceptualization, data duration, formal analysis, funding acquisition, investigation, methodology, project administration, validation, visualization, writing—original draft, writing—review and editing: Elizabeth A. Beilke. Conceptualization, funding acquisition, methodology, project administration, resources, supervision, writing—review and editing: Joy M. O'Keefe.

## CONFLICT OF INTEREST

None declared.

## Supporting information


Appendix S1

## Data Availability

Data (Beilke & O'Keefe, 2022) are available from Illinois Data Bank at https://doi.org/10.13012/B2IDB‐2455970_V1.
