# Supplementary material for: Bats reduce insect density and defoliation in temperate forests: An exclusion experiment
Source: Ecology. 2022 Dec 21;104(2):e3903. doi: 10.1002/ecy.3903 (PMC10078224; doi:10.1002/ecy.3903)
Supplement: Supplementary file 1 — Appendix S1 [file ECY-104-0-s001.pdf]

JOURNAL PUBLICATION CITATION: Beilke, E.A. and O'Keefe, J.M. 2022. Bats reduce insect density and defoliation in temperate forests: An exclusion experiment. *Ecology*.

Appendix S1. Supporting information for "Bats reduce insect density and defoliation in temperate forests: an exclusion experiment."

Files includes:

Supplementary Text
- Section S1
- Section S2

Supplementary Tables and Figures
- Figure S1
- Figure S2
- Figure S3
- Table S1

Supplementary Literature Cited

Authors:

Elizabeth A. Beilke
Department of Natural Resources and Environmental Sciences,
University of Illinois at Urbana-Champaign, Urbana, IL, USA; and
Center for Bat Research, Outreach, and Conservation,
Indiana State University, Terre Haute, IN, USA
lizz.beilke@gmail.com

Joy M. O'Keefe
Department of Natural Resources and Environmental Sciences,
University of Illinois at Urbana-Champaign, Urbana, IL, USA; and
Center for Bat Research, Outreach, and Conservation,
Indiana State University, Terre Haute, IN, USA
joyokeefe@gmail.com

Supplementary Text

Section S1. Camera trap surveys

In 2019 and 2020, we monitored the presence of non-bat fauna in each plot using two Stealth Cam STC-DS4K trail cameras (GSM Outdoors, Irving, TX). Cameras were set to photograph in bursts of three (8.0-megapixel resolution, 30 second timeout delay between activations). We deployed them simultaneously, one monitoring a bat-excluded plot and the other its paired control plot. We rotated these cameras between experimental units so that each control/treatment pair was monitored for at least two weeks during the study. This yielded a final sample size of 392 24-hour survey days. We recorded 21 visits by white-tailed deer (*Odocoileus virginianus*) and one visit by a wild turkey (*Meleagris gallopavo*).

Section S2. Acoustic surveys

In each experimental unit, we deployed an Anabat SD1 bat detector (Titley Scientific, Columbia, MO) which passively recorded bat acoustic activity for, on average, 36 nights in 2019, 47 nights in 2020, and 37 nights in 2020 (789 survey nights in total). All detectors were programmed to record from 30 minutes before sunset to 30 minutes after sunrise. We differentiated bat activity from noise using Bat Call Identification software version 2.8b (default settings; Bat Call Identification, 2021).

To investigate changes in bat activity (nightly call files) across years, we constructed a generalized linear mixed model with experimental unit id as a random intercept and a negative binomial family to account for overdispersion in the data. Our model included year (2018, 2019, or 2020) as a fixed effect (*bat activity ~ year + (1/unit ID)*). We fit our model using the

glmmTMB package (Brooks et al. 2017). We validated our model using the DHARMa package (Hartig 2021).

Within each experimental unit, we recorded 24 ± 1 bat calls per night (mean ± standard error). Bat activity was higher in 2018 than 2019 ($p = 0.030$) but was otherwise similar across years. Bats were active in all experimental units and were consistently present on the landscape (Figure S2).

Supplementary Tables and Figures

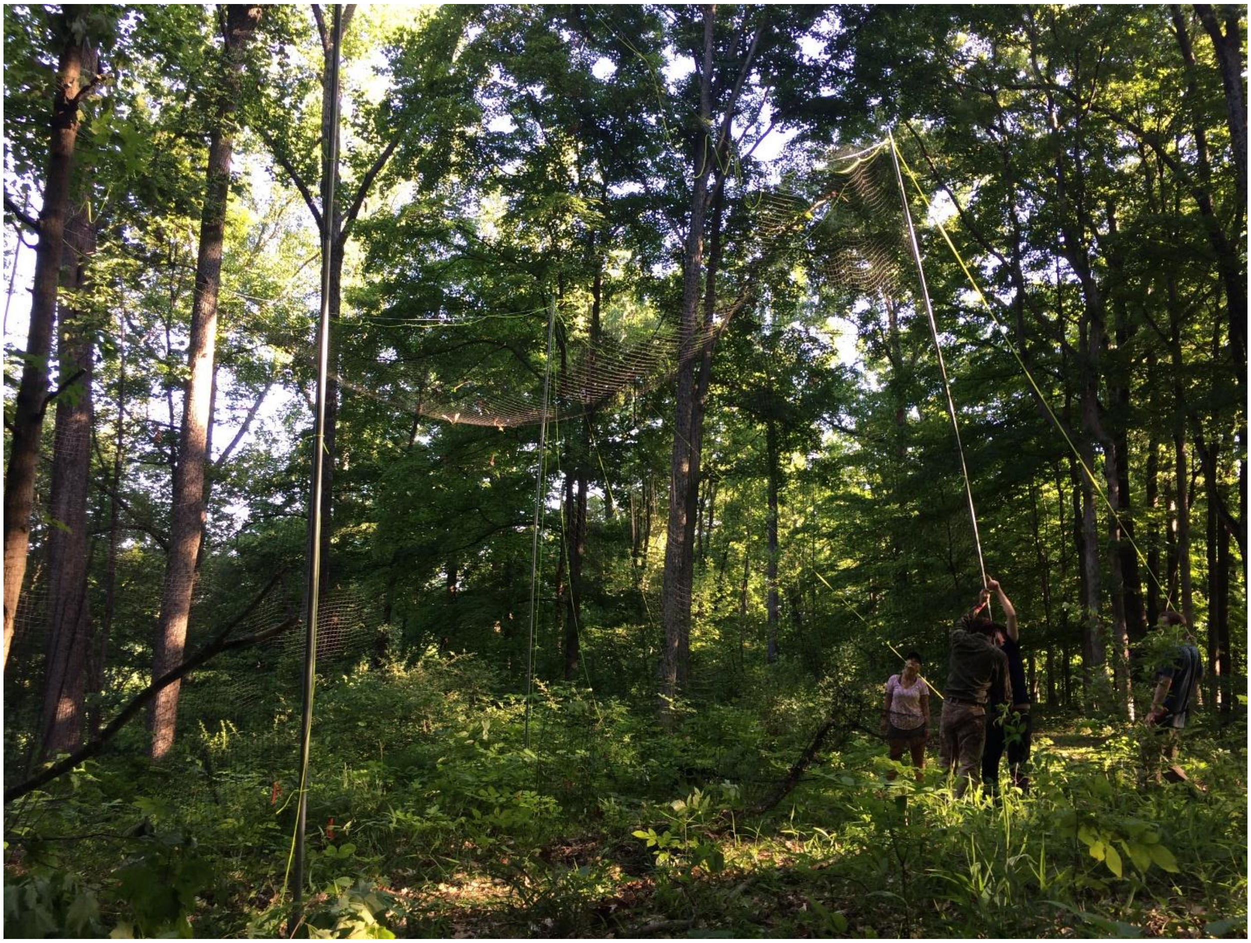

Figure S1. An exclosure being deployed at the start of the 2018 field season. Each plot consisted of four galvanized steel poles connected by a steel cable topline. Bat-excluded plots also had square-knot nylon mesh netting (4.44 cm) suspended from the exclosure frames using metal rings. Photo credit: Elizabeth Beilke.

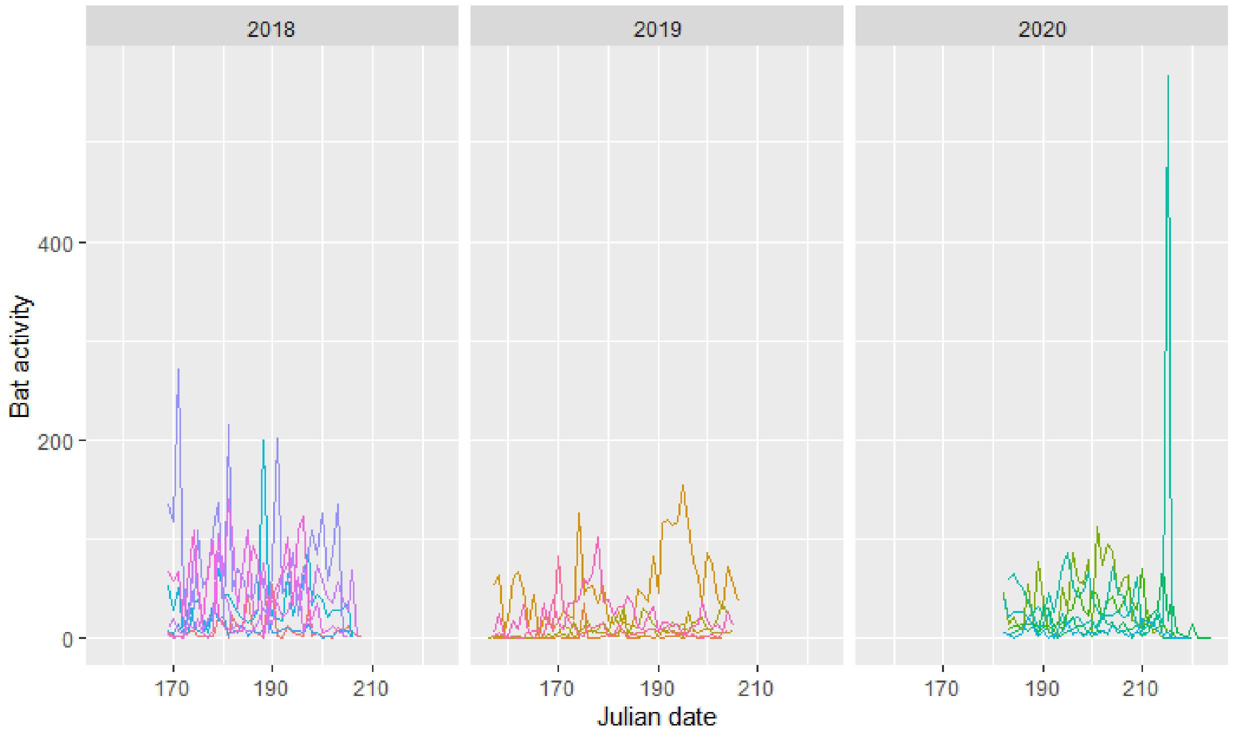

Figure S2. Seasonal patterns in bat activity (number of nightly acoustic call files) within experimental units. Experimental units were monitored for bat activity for a subset of the study duration (late May to mid-August; approx. Julian dates 144–227). Colors represent bat activity in different experimental units.

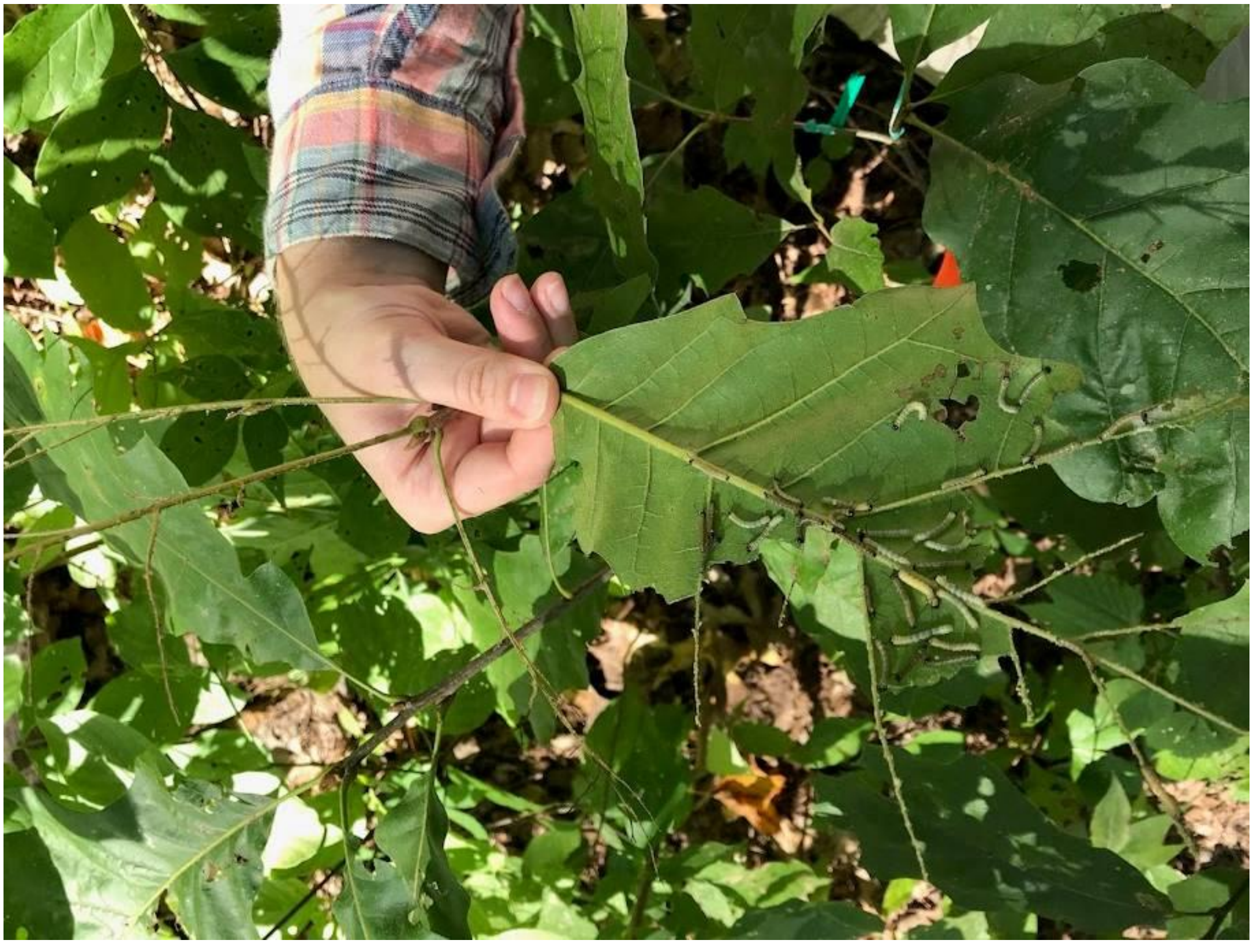

Figure S3. A study seedling in a bat-excluded plot, mostly defoliated by a large group of lepidopteran larvae. We made six times as many observations of groups of > 10 lepidopteran larvae on bat-excluded seedlings versus control seedlings. Photo credit: Elisabeth Kernan.

Table S1. Nocturnal animals that may have been incidentally excluded from bat-excluded plots and notes on their estimated abundance in Yellowwood State Forest, IN, USA and impact to study. Notes on abundance are collated from the personal experiences of Elizabeth A. Beilke, Danielle P. Williams[1], and Scott Haulton[2]. Impact was considered minimal for non-insectivores, non-defoliators, and terrestrial species never recorded in experimental units.

Taxonomic Class	Species	Detected by cameras in experimental units?	Notes on estimated abundance at study site	Projected impact to study
Aves	Barred owl (*Strix varia*)	Not detected	Common but not abundant	Minimal
	Eastern screech owl *(Megascops asio)*	Not detected	Uncommon and not abundant	Minimal
	Eastern whip-poor-will *(Antrostomus vociferus)*	Not detected	Common, but in decline. In Ontario, males defend territories that span 4–10 ha[3], but point count surveys from the study area reveal an estimated minimum density of 2.75 calling males per 1.6-km radius[2]. Given this density, it is likely any experimental unit was regularly exposed to 0–1 individual whip-poor-wills.	May have contributed to observed effects, though not nearly as much as bats.
Mammalia	Coyote (*Canis latrans*)	Not detected	Common but not abundant	Minimal
	Gray fox (*Urocyon cinereoargenteus*)	Not detected	Uncommon and not abundant	Minimal
	Raccoon (*Procyon lotor*)	Not detected	Commonly and abundant	Minimal
	Red fox (*Vulpes vulpes*)	Not detected	Uncommon and not abundant	Minimal
	Southern flying squirrel *(Glaucomys volans)*	Not detected	Common and abundant	Minimal
	Striped skunk (*Mephitis mephitis*)	Not detected	Common and abundant	Minimal
	Virginia opossum (*Didelphis virginiana*)	Not detected	Common and abundant	Minimal
	White-tailed deer (*Odocoileus virginianus*)	21 detections	Common and abundant	Minimal; no browse detected.

[1] Danielle P. Williams (Purdue University, Hardwood Ecosystem Experiment). Written communication. 2021
[2] Scott Haulton (Division of Forestry, Indiana Department of Natural Resources). Written communication. 2021
[3] Grahame E, Martin K, Gow E, Norris D. Diurnal and nocturnal habitat preference of Eastern Whip-poor-wills (*Antrostomus vociferous*) in the northern portion of their breeding range. Avian Conserv Ecol. 2021 Sep 22; 16(2).

Supplementary Literature Cited

Brooks, Mollie, E., Kasper Kristensen, Koen Benthem J.,van, Arni Magnusson, Casper Berg W., Anders Nielsen, Hans Skaug J., Martin Mächler, and Benjamin Bolker M. 2017. “GlmmTMB Balances Speed and Flexibility Among Packages for Zero-Inflated Generalized Linear Mixed Modeling.” *The R Journal* 9 (2): 378. https://doi.org/10.32614/RJ-2017-066.

Hartig, Florian. 2021. “DHARMa: Residual Diagnostics for Hierarchical (Multi-Level/Mixed) Regression Models.” https://CRAN.R-project.org/package=DHARMa.
